# Genetic Investigation of Complement Pathway Genes in Type 2 Diabetic Retinopathy: An Inflammatory Perspective

**DOI:** 10.1155/2016/1313027

**Published:** 2016-02-16

**Authors:** Ming Ming Yang, Jun Wang, Hong Ren, Yun Duan Sun, Jiao Jie Fan, Yan Teng, Yan Bo Li

**Affiliations:** ^1^Eye Hospital, The First Affiliated Hospital of Harbin Medical University, Harbin 150001, China; ^2^The Centre for Endemic Disease Control, Chinese Center for Disease Control and Prevention, Harbin Medical University, Harbin 150001, China; ^3^Department of Endocrinology, The First Affiliated Hospital of Harbin Medical University, Harbin 150001, China; ^4^Operating Room, The First Affiliated Hospital of Harbin Medical University, Harbin 150001, China

## Abstract

Diabetic retinopathy (DR) has complex multifactorial pathogenesis. This study aimed to investigate the association of complement pathway genes with susceptibility to DR. Eight haplotype-tagging SNPs of* SERPING1* and* C5* were genotyped in 570 subjects with type 2 diabetes: 295 DR patients (138 nonproliferative DR [NPDR] and 157 proliferative DR [PDR]) and 275 diabetic controls. Among the six* C5* SNPs, a marginal association was first detected between rs17611 and total DR patients (*P* = 0.009, OR = 0.53 for recessive model). In stratification analysis, a significant decrease in the frequencies of G allele and GG homozygosity for rs17611 was observed in PDR patients compared with diabetic controls (*P*_corr_ = 0.032, OR = 0.65 and *P*_corr_ = 0.016, OR = 0.37, resp.); it was linked with a disease progression. A haplotype AA defined by the major alleles of rs17611 and rs1548782 was significantly predisposed to PDR with increased risk of 1.54 (*P*_corr_ = 0.023). Regarding other variants in* C5* and* SERPING1*, none of the tagging SNPs had a significant association with DR and its subgroups (all *P* > 0.05). Our study revealed an association between DR and* C5* polymorphisms with clinical significance, whereas* SERPING1* is not a major genetic component of DR. Our data suggest a link of complement pathway with DR pathogenesis.

## 1. Introduction

Diabetes mellitus (DM) is reaching an alarming proportion worldwide, as is known that DM has a complex multifactorial pathogenesis. The devastating complications of diabetes are the macro- and microvascular diseases [1, 2]. Of them, diabetic retinopathy (DR) is the most common microvascular complication and is a leading cause of blindness across the globe [3]. To date, many environmental and clinical factors have been proposed to confer risk of DR development, such as prolonged duration of diabetes, alteration of glucose metabolism, and poor glycemic control [4]. Additionally, genetic predisposition, independent of the above-mentioned factors, has been found to contribute to DR pathology; the evidence comes from the observation of disease aggregation among family members and multiple DR-associated genes identifications [5–7]. So far, the exact pathogenesis of DR is still unclear and is known to be involved in several physiopathologic pathways, such as angiogenesis factors, oxidative stress, apoptosis, and protein kinase C (PKC) [8–10]. More recent evidence depicting DR as a retinal disease associated with inflammation has drawn special attention and garnered great research interests [11–13].

Complement system is an important component of innate immunity and involved in the modulation of several immune and inflammatory responses. The complement system can be divided into classical, lectin, and alternative pathway; activation of the system is tightly regulated by complement factors; disruption of complement regulation can lead to several distinct downstream inflammatory actions* en route to* the pathogenesis of DR [14, 15]. Evidence for the link comes from the observation of increased expression of several complement factors in DR patients; these factors included C1 inhibitor (C1INH, also known as serpin peptidase inhibitor, clade G,* SERPING1*), C5, factor H (CFH), and factor B (CFB) [16, 17]. In our previous studies, genetic variants in the* CFH* and* CFB* genes, both involved in complement alternative pathway, have been evaluated and identified as susceptibility genes for DR [18]. Moreover,* CFH* and* CFB*, as well as other complement pathway genes, have also been found to be associated with a range of inflammatory diseases [19].

Therefore, a genetic study focused on other complement genes was designed with a view to elucidating the involvement of complement system in DR development. Two complement genes,* SERPING1* and* C5*, involved in the classical pathway and in the central part of complement cascade, respectively, were selected for evaluation. Furthermore, stratification by DR stage and genotype-phenotype correlation analysis were also performed to identify these factors associated with prognosis and clinical features.

## 2. Materials and Methods

### 2.1. Study Participants

The study protocol was approved by the Ethics Committee on Human Research, Harbin Medical University. The study procedures were conducted in accordance with the tenets of the Declaration of Helsinki. Written informed consent was obtained from all study subjects after explanation of the nature of the study. All study subjects were Han Chinese recruited from the First Affiliated Hospital of Harbin Medical University.

All patients received complete ophthalmic examinations and clinical information collection, including corrected visual acuity, slit-lamp biomicroscopy, fundoscopic examination, age, gender, progression time from diabetes to DR, body mass index (BMI), HbA1c level, smoking status, and presence of hypertension and hyperlipidemia, as well as insulin application. The study involved 570 unrelated individuals with type 2 diabetes mellitus (T2DM); patients with type 1 diabetes, gestational diabetes, or maturity-onset diabetes were excluded from the study. The diagnosis of T2DM was based on World Health Organization criteria [20]. Of the group, 295 patients were diagnosed with DR (156 [52.9%] PDR and 139 [47.1%] NPDR); 275 subjects without DR but with type 2 diabetes duration of more than 10 years were considered as DM controls. The stage of DR was determined according to the Early Treatment Diabetic Retinopathy Study (ETDRS) criteria [21]. People with any systemic inflammation diseases, or any other ocular disorders such as age-related macular degeneration (AMD), glaucoma, or retinal venous occlusion, were also excluded.

### 2.2. SNP Selection and Genotyping

We adopted a haplotype-tagging SNP approach and obtained the tagging SNPs across the targeted regions, from the International HapMap Project for the Chinese Han Beijing (CHB) population (http://hapmap.ncbi.nlm.nih.gov/, HapMap Genome Browser). Two SNPs (rs1005511 and rs3824988) from* SERPING1* and six from* C5* (rs12237774, rs2269066, rs17611, rs1548782, rs10985126, and rs1017119) were selected by the tagger-pairwise method with *r* ^2^ and MAF (minor allele frequency) values greater than 0.8 and 0.10, respectively. Genomic DNA was extracted from whole blood using a QIAamp Blood Kit (Qiagen, Hilden, Germany) according to the protocol. All the SNPs were genotyped by* Taq*Man SNP Genotyping Assays (Applied Biosystems Inc., Foster City, CA) in the LightCycler® 480 Real-Time PCR System (Roche, Switzerland) according to the manufacturer's instructions.

### 2.3. Statistical Analysis

Hardy-Weinberg equilibrium (HWE) of individual SNP was tested by *χ* ^2^ test. Allelic and genotypic association of each SNP was calculated by using *χ* ^2^ test or Fisher exact test. Dominant and recessive models were also applied to investigate the disease association with regard to the minor allele. The odds ratios (OR) and 95% confidence intervals (CI) were calculated. Pairwise linkage disequilibrium (LD, *D*′) between polymorphisms and expectation-maximization- (EM-) based haplotype association analysis were assessed using the Haploview software. Student's *t*-test and *χ* ^2^ test were used to compare continuous clinical data and categorical variables, respectively. Stratification analysis based on DR stage (NPDR and PDR) was also performed. *P* < 0.05 was considered as statistically significant. *P* values were corrected by Bonferroni test (*n* = total number of SNPs) or permutation test in Haploview software.

## 3. Results

In our study, a total number of 570 unrelated individuals with T2DM were recruited, comprising 295 DR patients and 275 DM controls. Since we aimed to recruit T2DM patients without DR as controls, the mean duration of disease was longer than that of the DR group so as to largely rule out late-onset DR (*P* < 0.01). The proportions of hyperlipidemia and insulin application were higher in DR group than that in DM controls (*P* = 0.042 and *P* < 0.001, resp.). No significant differences in other clinical features were observed between two groups (Table 1).

Two haplotype-tagging SNPs in* SERPING1* and six haplotype-tagging SNPs in* C5* were selected, which capture over 90% of all alleles across their corresponding locus with a MAF larger than 0.10 and a mean *r* ^2^ of 0.80 in the HapMap Chinese Han population. The genotype frequencies of the eight selected SNPs followed the HWE in all subjects. For* C5*-rs17611, there was an obvious trend towards lower proportions of G allele and GG homozygosity in DR patients than DM controls (*P* = 0.056, OR = 0.79, 95% CI = 0.62–1.0; *P* = 0.009, *P*_corr_ = 0.072, OR = 0.53, 95% CI = 0.33–0.86, resp.), but the associations either were marginal or could not remain after adjustment for multiple testing. For other SNPs, no significant associations were detected with DR in any genetic models (Table 2).

Among the 295 DR patients, 139 (47.1%) were NPDR and 156 (52.9%) were PDR; stratification analysis by the DR stage was performed. In PDR patients, significant lower frequencies of G allele and GG homozygosity for* C5*-rs17611 were found compared to that in DM group even after multiple testing correction (*P*_corr_ = 0.032, OR = 0.65, 95% CI = 0.48–0.87; *P*_corr_ = 0.016, OR = 0.37, 95% CI = 0.19–0.71, resp.), implying a protective effect; such difference was not observed in NPDR patients (Table 3). For other SNPs, no significant differences in the allelic or genotypic frequencies were found in either NPDR or PDR subtypes compared with DM controls.

**Figure 1:**
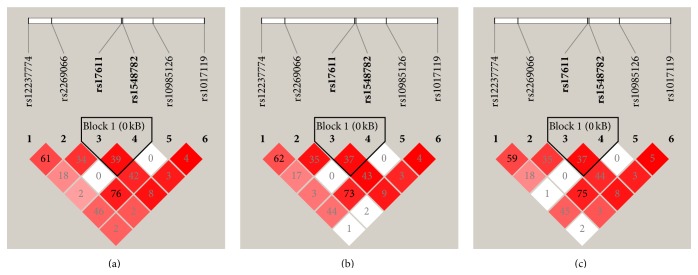
Linkage disequilibrium (LD) structure of the* C5* locus for DR (a), NPDR (b), and PDR (c). LD was measured using data from all controls and total DR and its subtypes. The haplotype block was defined by the confidence interval method implemented in the Haploview software. The LD (*r* ^2^) between any two SNPs is listed in the cross cells. DR: diabetic retinopathy; NPDR: nonproliferative diabetic retinopathy; PDR: proliferative diabetic retinopathy.

Pairwise LD analysis showed that two* SERPING1* tagging SNPs were included in one haplotype block in NPDR, PDR, and total DR patients. Three groups showed a similar distribution of haplotype. No haplotype was significantly associated with any group (all *P* > 0.1, Table 4). Regarding* C5*, LD analysis revealed one haplotype block in three groups including SNPs rs17611 (the most significant finding) and rs1548782 (Figure 1). The haplotype AA, defined by the two SNPs, showed a significant risk for PDR patients (*P* = 0.004, permutation *P* = 0.023; OR = 1.54, 95% CI = 1.15–2.06). No significant haplotype association was detected among the other two comparisons (Table 5).

**Figure 2:**
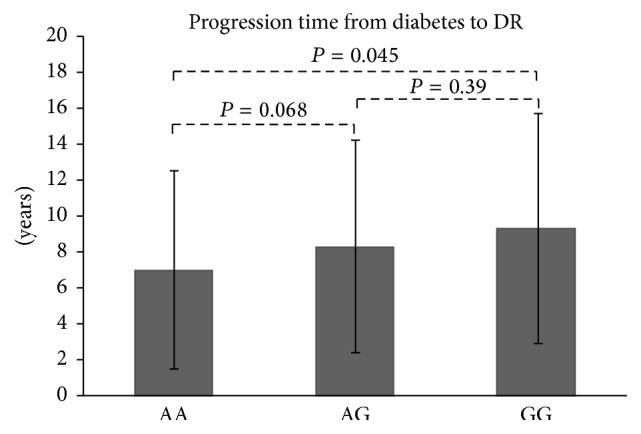
The average time of progression from diabetes to DR according to genotype. AA: 7.0 ± 5.5; AG: 8.3 ± 5.9; GG: 9.3 ± 6.4 (years).

Considering the significance of* C5*-rs17611 in this study, correlations of the specific genotype with clinical features were evaluated in total DR patients. The results showed that DR patients carrying protective rs17611 GG genotype would present a delayed progression from DM to DR onset compared with patient carrying AA genotype (9.3 ± 6.4 versus 7.0 ± 5.5, *P* = 0.045; Figure 2); no significant difference for other clinical features was detected among different genotype carriers.

## 4. Discussion

In this study, we performed a haplotype-tagging SNP analysis of two complement pathway genes,* SERPING1* and* C5*, in T2DM and DR patients. Our results demonstrated that* C5*-rs17611 was significantly associated with DR, particularly conferred to the PDR susceptibility; this functional variant also linked with certain clinical significance. Moreover, a haplotype conferring an increased risk for PDR was also detected. In contrast, none of the SNPs in* SERPING1* were significantly associated with DR and its subtypes. These findings together suggest that* SERPING1* is not a disease gene for DR, but* C5* is likely to be a susceptibility gene for DR in Chinese patients. To our knowledge, this is the first genetic study to investigate* SERPING1* and* C5* genes in DR patients. Over the past decade, great achievements have been made in elucidating the genetic background of the disease; so far, more than 30 DR-associated genes involved in different metabolic mechanisms and functional pathways have been reported [22, 23]. Our previous study has successfully identified two complement alternative pathway genes,* CFH* and* CFB*, which were associated with DR development [18]. Results of this study enrich our knowledge of the genetic architecture of DR and the involvement of each complement pathway in DR pathogenesis. In addition, we also found that the proportions of insulin application were higher in DR group than that in DM controls; it was supposed that these DM patients without complications have a relatively good metabolic control, which may explain the lower frequency of individuals with insulin therapy.

As described above, the complement system is a key component of innate immunity, consisting of a large family of membrane-bound proteins that are critical for protection against bacterial infection and immune complex deposition. Uncontrolled complement activation is considered an important contributor in the pathogenesis of DR [15]. C5, being the first of many components of the terminal pathway, mediates many potent inflammatory events and plays a major role in the complement system. In the cascade, a critical event is the cleavage of C5 into fragments of C5a and C5b, as well as the subsequent formation of MAC (C5b-9) which is involved in cytolysis, cell activation, and production of inflammatory mediators [24].* In vitro* study has revealed that C5a treatment induced increased production of several inflammatory cytokines, such as MCP-1, IL-6, IL-8, and VEGF, from retinal pigment epithelial cells [25, 26]. In clinical study, C5b-9 deposition was detected on the endothelial surface of retinal vessels in eye donors with diabetes; vitreous concentration of C5a increased significantly in PDR patients compared with controls [14, 27]. Furthermore,* C5* gene has been found to affect susceptibility to several inflammatory conditions, including AMD, rheumatoid arthritis, and renal allograft outcomes [28–30]. In the present study,* C5*-rs17611 was found to be associated with PDR patients; meanwhile, rs17611 was also found to be associated with periodontitis and the GG genotype was linked with increased C5 levels in patients with rheumatoid arthritis [29, 31]. The change of rs17611 A>G nucleotide results in the synthesis of Valine instead of Isoleucine; a functional analysis on rs17611 showed that individuals homozygously expressing the risk s17611 allele exhibit increased C5a and decreased C5 in plasma, evidence of increased C5 turnover; this structural change might alter the rate of C5 cleavage and explain its association with inflammatory diseases [29].

Component 1 inhibitor gene (*SERPING1*) encoding C1INH is a key regulator in classic and lectin complement pathway and involved in the development of several immune-related diseases. In addition, C1INH was also found to be expressed in both retinal and retinal pigment epithelium (RPE) layers [32–34]. Based on the evidence,* SERPING1* was considered as a candidate gene for DR. But, in our study, no association between* SERPING1* polymorphisms and DR was found, even stratified by DR stage or considered clinical features. The results suggested that* SERPING1*, and the gene-involved classical pathway, might not contribute significantly to the risk of DR. Further studies to determine the biologic roles of these polymorphisms and the haplotype in DR are still warranted; additionally, it would be better to include healthy controls in this study to fully reflect the disease association.

In summary, this study first demonstrated that* C5* rs17611 is a susceptibility locus for DR and particularly predisposes to PDR subtype with clinical significance. The complement classical pathway gene,* SERPING1*, may confer no or limited risk for DR development. Together with our previous findings, our results help to further enrich the growing understanding of genetic spectrum of DR and clarify the involvement of each complement pathway in DR pathogenesis from molecular perspectives.

## Figures and Tables

**Table 1:** Characteristics of the study subjects.

Characteristic	DR (*n* = 295)	DM (*n* = 275)	*P* value
Age (years)	55.9 ± 13.2	56.3 ± 7.6	0.66
Gender (F/M)	151/144	153/122	0.29
Duration of diabetes (years)	13.1 ± 9.4	18.1 ± 6.7	<0.01
Duration of DR (years)	5.3 ± 3.9	—	N/A
Progression time from diabetes to DR (years)	7.9 ± 5.8	—	N/A
HbA1C (%)	8.0 ± 1.6	7.9 ± 1.9	0.50
BMI (kg/m^2^)	24.0 ± 5.7	24.1 ± 4.4	0.82
Hypertension (%)	73.2	66.9	0.10
Hyperlipidemia (%)	30.8	23.3	0.042
Smoking (%)	13.9	16.0	0.48
Insulin therapy (%)	46.8	26.2	<0.001
Family history of diabetes (%)	26.1	21.5	0.19

*P* values were compared by *χ* ^2^ or Student's *t*-test, and *P* < 0.05 was considered statistically significant. DR: diabetic retinopathy; DM: diabetes mellitus; HbA1c: glycosylated hemoglobin; BMI: body mass index.

**Table 2:** Comparison of genotype and allele frequencies of *SERPING1 *and *C5* polymorphisms in DR patients and DM controls.

SNP ID	Minor allele	Allele distribution (%)			*P* value	Odds ratio (95% CI)	Genotype distribution (%)		*P* value	Odds ratio (95% CI)
		DR (*n* = 590)		DM (*n* = 550)			DR (*n* = 295)	DM (*n* = 275)		
C1INH (*SERPING1*)										
rs1005511	G	G	137 (23.2)	125 (22.7)	0.84		173/107/15	165/95/15	0.74^†^	
		A	453 (76.8)	425 (77.3)					0.84^‡^	
rs3824988	C	C	71 (12.0)	57 (10.4)	0.37		228/63/4	220/53/2	0.43^†^	
		T	519 (88.0)	493 (89.6)					0.68^‡*∗*^	

*C5*										
rs12237774	T	T	107 (18.1)	109 (19.8)	0.47		199/85/11	175/91/9	0.34^†^	
		C	483 (81.9)	441 (80.2)					0.82^‡*∗*^	
rs2269066	T	T	121 (20.5)	120 (21.8)	0.59		187/95/13	165/100/10	0.41^†^	
		C	469 (79.5)	430 (78.2)					0.64^‡^	
rs17611	G	G	210 (35.6)	226 (41.1)	0.056	0.79 (0.62–1.0)	117/146/32	100/124/51	0.42^†^	0.53 (0.33–0.86)
		A	380 (64.4)	324 (58.9)					0.009^‡^ (0.072)	
rs1548782	T	T	119 (20.2)	112 (20.4)	0.94		191/89/15	172/94/9	0.59^†^	
		A	471 (79.8)	438 (79.6)					0.28^‡^	
rs10985126	C	C	136 (23.1)	134 (24.4)	0.60		170/114/11	160/96/19	0.89^†^	
		T	454 (76.9)	416 (75.6)					0.089^‡^	
rs1017119	C	C	73 (12.4)	78 (14.2)	0.37		225/67/3	202/68/5	0.44^†^	
		T	517 (87.6)	472 (85.8)					0.49^‡*∗*^	

Data are the number of subjects (% of the total group). DR: diabetic retinopathy; DM: diabetes mellitus; ^*∗*^Fisher exact test; ^†^ *P* value for dominant model; ^‡^ *P* value for recessive model. The genotype distribution was showed as major allele homozygous/heterozygous/minor allele homozygous.

**Table 3:** Comparison of genotype and allele frequency of *C5*-rs17611 in DR and DM stratified by disease severity.

SNP ID	Genotype/allele	NPDR (*n* = 139)	PDR (*n* = 156)	DM (*n* = 275)	NPDR versus DM		PDR versus DM	
					*P* value	Odds ratio (95% CI)	*P* value	Odds ratio (95% CI)
rs17611	GG	20 (14.4)	12 (7.7)	51 (18.5)	0.51^†^	1.15 (0.75–1.78)	0.062^†^	0.68 (0.46–1.02)
	AG	73 (52.5)	73 (46.8)	124 (45.1)	0.29^‡^	0.74 (0.42–1.30)	0.002^‡^ (0.016)	0.37 (0.19–0.71)
	AA	46 (33.1)	71 (45.5)	100 (36.4)	0.62^#^	0.85 (0.46–1.59)	0.001^#^ (0.008)	0.33 (0.17–0.67)
	G	113 (40.6)	97 (31.1)	226 (41.1)	0.90	0.98 (0.73–1.32)	0.004 (0.032)	0.65 (0.48–0.87)
	A	165 (59.4)	215 (68.9)	324 (58.9)				

Data are the number of subjects (% of the total group). NPDR: nonproliferative diabetic retinopathy; PDR: proliferative diabetic retinopathy; DM: diabetes mellitus. ^†^ *P* value for dominant model; ^‡^ *P* value for recessive model; ^#^ *P* value for codominant model.

**Table 4:** Haplotype association of *SERPING1* gene with DR and its subtypes.

Haplotype rs1005511- rs3824988	Frequency				Association (*P* value)		
	Total DR	NPDR	PDR	DM	DR versus DM	NPDR versus DM	PDR versus DM
A-T	0.76	0.79	0.74	0.77	0.79	0.51	0.31
G-T	0.12	0.11	0.13	0.13	0.60	0.38	0.99
G-C	0.12	0.10	0.13	0.10	0.42	0.96	0.19

**Table 5:** Haplotype association of *C5* gene with DR and its subtypes.

Haplotype rs17611- rs1548782	Frequency				Association (*P* value) (permutation test)		
	Total DR	NPDR	PDR	DM	DR versus DM	NPDR versus DM	PDR versus DM
A-A	0.64	0.59	0.69	0.59	0.064	0.94	0.004 (0.023)
G-T	0.20	0.23	0.17	0.20	0.88	0.29	0.21
G-A	0.16	0.17	0.15	0.21	0.025 (0.11)	0.21	0.019 (0.09)

NPDR: nonproliferative diabetic retinopathy; PDR: proliferative diabetic retinopathy; DM: diabetes mellitus. *P*_corr_ association analysis results from permutation test (iterations 10,000).
